# Supplementary material for: Are carbon emissions trading and green financial instruments synergistic? -Comprehensive quantitative research based on content analysis
Source: PLoS One. 2024 Mar 7;19(3):e0298601. doi: 10.1371/journal.pone.0298601 (PMC10919601; doi:10.1371/journal.pone.0298601)
Supplement: S1 Data — (ZIP) [file pone.0298601.s001.zip › Data/地级市Z_F工作报告.zip!/╣π╬≈/╟╒╓▌╩╨/└┤╫╘╣1⁄2╓┌║┼í╛┬φ┐╦╩2╛▌═°í┐_2014.docx]

各位代表：

现在，我代表市人民政府向大会报告工作，请予审议，并请市政协委员和其他列席会议的同志提出意见。

2013年工作回顾

过去的一年，是我市经济发展较为困难的一年，也是我们振奋精神、锐意进取的一年。在李克强总理考察钦州讲话精神鼓舞下，在市委坚强领导下，全市上下认真贯彻中央和自治区的一系列决策部署，牢牢把握“稳中求进、开拓进取、凸显成效”的工作基调，深入实施五大方略，积极应对各种挑战，立足破难题、打基础、增后劲，以昂扬饱满的热情克难攻坚、顽强拼搏，继续保持了经济社会平稳发展。预计全市生产总值完成750亿元，同比增长9%；规模以上工业总产值1115亿元，增长2%；固定资产投资560亿元，增长35%；地方公共财政预算收入42亿元，增长25%；城镇居民人均可支配收入23760元，增长10%；农民人均纯收入8140元，增长14%。

这一年，我们牢牢把握产业振兴这一主攻方向，坚定不移推进重要产业发展工程，努力做大经济总量，提升发展质量。

坚持各类产业同步推进，促进产业集聚化布局、规模化发展。启动实施“千百亿产业三年攻坚工程”，加快壮大临港产业集群，金桂纸业、天恒石化二期、澄星磷化工等竣工投产，中船大型海工修造及保障基地、国投电厂二期启动建设，嘉华钛白粉、华汇镍业等进展顺利。启动实施“县域工业壮大工程”，完善县域工业发展壮大的政策措施，突出抓好民营经济和中小微企业发展，建成灵山天山微电子三期、浦北裕源产学研基地等项目，培育新增规模以上企业34家，县域规模以上工业总产值480亿元、增长46%，成为推动经济增长的重要支撑。实施“高新产业培育工程”，依托高新区和其他开发园区，着力抓好专利倍增和平台搭建工作，高新技术企业总数达到18家，建成一批自治区级、市级工程技术研发中心。实施“筑巢引博”行动，引进3个领军型创业创新团队。着力抓好现代服务业发展，出台落实建筑业、房地产业、交通运输业扶持政策。完善区域性商贸项目布局，东盟商贸城、东盟农产品大市场等一批骨干项目开工建设，大润发实现开业，千年古陶城加快推进，成功举办第二届中国·钦州国际蚝情节，社会消费稳步扩大，旅游业较快增长。金融业持续发展，新引进2家保险公司，成立4家小额贷款公司，浦北国民村镇银行开业。大力推进“特色农业提升工程”，粮食生产单产破历史记录，超额完成粮食生产任务。积极抓好“菜篮子”工程，市区农贸市场升级改造稳步推进。浦北成为全国首批林下经济基地。新增农民专业合作社109家，市级以上重点龙头企业10家。台湾农民创业园、“中国龟谷”加快建设。农村基础设施建设得到加强。

这一年，我们充分发挥港口这一核心资源优势，坚定不移推进港城互动，努力增强港口和城市辐射带动作用。

坚持以港口为龙头、城市为依托，推动港城联动发展，全市城镇化率达到44%。重点推进亿吨大港建设，30万吨级主航道竣工，30万吨级油码头完成水工工程，建成新天德7万吨级散杂货码头。集疏运体系加快完善，六钦、钦崇高速公路通车，南钦、钦北、钦防高铁开通营运，黎钦铁路复线完成铺轨，钦灵一级公路加快建设。新开通钦州港至宁波、珠海和台湾高雄等直航航线，港口吞吐量突破6000万吨，其中集装箱吞吐量60万标箱。滨海新城建设展现新形象，扬帆大道南延长线全线贯通，白石湖片区主干路网全面施工，北部湾国际人才创业基地、东盟生态园加快建设，白石湖公园建成开放。以城市环境综合整治为突破口，统筹推进创“三城”工作。重点整治市区“四场一点”，实施金海湾东西大街改造，完成城市中心大货车、拖拉机限行整治。加大控违查违力度，加快打通城市“断头路”。林湖公园一期建成开园，中山公园改扩建加快推进。突出抓好县城新区和重点中心镇建设，推进“镇改办、村改居”试点。

这一年，我们创新利用各类特殊政策和合作平台，坚定不移推进开放带动、投资拉动，努力增创开放发展新优势。

积极依托国家级、自治区级和一批特色产业园区，加快构建园区经济体系。各级各类开发区产业集聚效应日益凸显。全力支持中马钦州产业园区加快发展，完成中马开发合资公司组建，争取到国家连续三年每年给予补助资金8亿元。全力支持保税港区二、三期建设，建成汽车滚装码头等一批功能设施，入园企业达到132家。全力支持对内开放合作，川桂临海产业园建设稳步推进。对外贸易稳步发展，磷酸出口基地成为首批“广西外贸转型升级专业型示范基地”。启动以钦州为基地建设中国－东盟港口城市合作网络，海上互联互通成为对外合作新亮点。千方百计引项目、抓服务、促投资。集中出台招商引资“1个意见5个办法”，整合组建9个招商小分队开展专业招商，实行全员招商和领导带头招商。全力开展“民企入桂”对接活动，成功引进华谊煤基多联产、凯富能源等一批重大项目。全年招商引资实际到位资金660亿元，增长20%。组织实施“千项工程大会战”和征地搬迁“百日大会战”，着力破解土地征拆难题，切实强化市领导和部门投资责任制，建立完善37个重大项目建设指挥部，全年固定资产投资增速居全区第二位，工业投资占固定资产投资比重48%。坚持以开放倒逼改革，进一步理顺城区管理体制，实行管理重心下移，下放征地搬迁、环卫管理等权限。制定并实施国有企业“分类指导、打包改革”方案。推进扩权强县工作，进一步下放审批权限。完善财税联席会议制度，合力推进“财源经济发展三年倍增计划”。做好平台公司融资和债务管理工作，白海豚投资置业股份有限公司成功在新三板上市，实现直接融资零突破。

这一年，我们紧紧扣住保障和改善民生这一根本出发点，全面发展社会事业，努力增进人民福祉、共享美好生活。

继续大力加强社会保障工作，率先在全区开展城乡居民大病保险试点，率先在全区建成“五险一金”信息集中管理系统，率先在全国发行首张载有金融功能的社会保障卡，稳步推进公立医院综合改革试点。就业形势保持稳定，社会保险覆盖面进一步扩大，保障性安居工程建设任务全面完成。提高城乡低保标准，城区和两县居民分别提高到每人每月400元和360元；农村低保标准每人每年由1500元提高到2300元。继续深化“携手共创平安和谐家园”活动，试点社区网格化管理，加强社会管理综合治理。预防青少年违法犯罪“钦南模式”在全国推广。完善矛盾调解机制，领导干部接访、带案下访和包案制度有效落实。强化安全监管和隐患大排查，切实抓好重点领域安全工作。推进食品药品安全监管体制改革，加强防灾减灾体系建设，应急管理体系进一步完善。强化绿色发展理念，继续推动“园林生活十年计划”和“绿满八桂”活动，加强钦江流域和茅尾海综合治理，即将建成城区应急水源项目。加强海洋生态环境保护，强化节能减排，主要污染物排放总量完成自治区下达任务。以“美丽钦州·清洁乡村”活动为抓手，逐步建立农村保洁长效机制。继续加大社会事业投入，为民办10件实事工程完成年度投资计划，有效解决了一批民生难题。加快北部湾大学和中小学标准化建设，新建和改扩建一批中小学、农村幼儿园。文化惠民活动扎实开展，全民健身活动取得新进展。灵山县获全国特色文化产业示范县，浦北县、钦南区成功申报自治区级文化产业示范县。全面做好人口计生工作，落实优抚安置政策，深入开展双拥共建活动。支持工会、共青团、妇联等人民团体工作，海关、出入境检验检疫、海事、边检等口岸联检部门在对外开放中发挥了重要作用，税务、人民银行、银监、工商、质检、供电、烟草、通信、邮政、盐务等在服务地方发展中做出了积极贡献，民族、宗教、外事、侨务、农机、供销、新闻出版、广电、统计、贸促、人防、海防、科普、地震、气象、水文、档案、扶贫、地方志、老龄、残疾人和关心下一代等事业得到进一步加强。

这一年，我们始终抓住政府自身建设这一组织保障，抓作风提效能，强执行重服务，努力树立政府良好形象。

我们大力弘扬“自强实干、融和共赢”的新时期钦州精神，秉承“团结、开拓、务实、高效”的工作理念，自觉树立和维护风清气正、廉洁高效、亲民法治的政府形象。深入贯彻落实中央“八项规定”，坚决反对和纠正“四风”。坚持向人大报告和向政协通报制度，自觉接受人大法律监督、政协民主监督和社会监督，及时办理市人大代表建议和政协提案293件。建立完善“市长企业接待日”、市长热线制度，多渠道听民声、察民情，主动问计于民，重点解决企业和群众关心的热点难点问题。深化行政审批制度改革，强化重大事项和重点工程的政务督查和效能监察，突出整治“中梗阻”。加强党风廉政建设，强化行政监察和审计监督，惩防体系建设取得明显成效。

各位代表，过去一年，在国内外经济形势复杂多变、我市经济下行压力异常加大的背景下，全市干部群众保持了良好的精神状态，敢于直面挑战，敢于勇挑重担，敢于克难攻坚，思想上同心同向，工作上务实进取，困境中求突破，发展中现亮点，进一步夯实了经济社会发展的良好基础。取得这些成绩实属不易，这归功于自治区党委、政府和市委的正确领导，归功于市人大、政协、各民主党派和社会各界的共同努力，归功于驻钦各部队、驻钦各单位的鼎力支持，归功于全市人民的团结拼搏。在此，我代表市人民政府，向广大人民群众和所有关心支持钦州发展的各界人士和朋友们，致以崇高的敬意和衷心的感谢！

同时，我们也清醒地看到，去年我市主要经济指标出现下滑，集中暴露了经济社会发展中的深层次矛盾和问题。一是经济总量仍然偏小，重大支撑项目不多，民营经济不发达，经济增长的基础还比较脆弱；二是港口带动作用仍然不强，集疏运体系不完善，配套基础设施滞后，港航服务能力较弱；三是产业仍然处于培育期，工业结构相对单一，土地、资金、劳动力等瓶颈制约仍然十分突出；四是财政收入结构不合理，地方可用财力低，统筹城乡发展任务十分艰巨；五是政府依法行政和治理能力有待进一步提高等。对于这些问题，我们将采取更加有力的举措加以解决。

2014年工作总体要求和目标任务

2014年是全面贯彻落实党的十八届三中全会精神的开局之年，也是我市推进“五年跨越、十年巨变”的关键之年。综合判断，国内外经济形势依然错综复杂，经济发展面临诸多困难和挑战，我市正处在爬坡过坎、不进则退的临界点上，各项发展任务十分艰巨。但国内经济长期向好的趋势没有改变，北部湾开放开发的政策效应没有改变，我市经过多年发展孕育的巨大发展潜力和后发优势没有改变，特别是随着西南中南地区开放发展新的战略支点的打造、全面深化改革工程的深入推进，各级各方面对钦州发展要求更高、期待更多、支持更大，全市人民思富盼强、跨越发展的愿望更加强烈。不进则退，慢进也是退。我们一定要坚定必胜信心，强化机遇意识和责任意识，唱响加快发展、实干兴市的主旋律，奋力开创全市经济社会发展新局面！

今年政府工作的总体要求是：全面贯彻落实党的十八届三中全会和中央、自治区经济工作会议以及市委四届四次全会精神，紧紧围绕主题主线，围绕开放发展新的战略支点的新要求，以稳中求进、提速提质、改革创新、彰显成效为总基调，乘东风促改革，转职能促发展，强作风显成效，以扩大开放为引领，以“三个年”活动为抓手，转作风，强服务，全力推进新型工业化、新型城镇化和农业现代化，促进经济快速发展、社会和谐稳定，为提前全面建成小康社会打下坚实基础。

经济社会发展的主要预期目标是：生产总值增长11%；财政收入增长10%，其中地方公共财政预算收入增长20%；规模以上工业总产值增长28%；固定资产投资增长25%；社会消费品零售总额增长14%；外贸进出口总额增长14%；港口吞吐量增长10%；城镇居民人均可支配收入增长11%，农民人均纯收入增长13%；城镇登记失业率控制在4.2%以内；居民消费价格指数控制在4%以内；人口自然增长率控制在8.6‰以内。围绕上述目标要求，今年重点要做好以下九个方面的工作。

一、深入开展“企业服务年”活动，全力推进工业经济跨越发展。全面推进工业跨越发展“1112工程”，牢固树立“亲商、安商、富商”的发展理念，坚决打好服务企业组合拳，为工业强市奠定坚实基础。

全力服务临港产业集聚发展。以临港工业园区为依托，重点推进石油化工、装备制造、粮油加工、电力能源以及冶金、造纸等支柱产业发展。加快推进园区基础设施配套，强化园区之间水电路网衔接，突出抓好产业专用码头、航道、管网体系建设。围绕支柱产业发展完善产业链条，不断强化临港产业集群的带动效应。继续开展“市长企业接待日”活动，启动“市长联系卡”活动，完善领导联系重点企业制度，强化职能部门协作和服务支持，及时解决企业生产经营中存在的突出矛盾和问题。实施“金龙头”企业培育工程，确保中石油、中粮、金桂等一批骨干企业稳产达产，创造条件支持更多企业做大做强，努力把钦州港经济技术开发区打造成为千亿元经济技术开发区。

全力服务县域工业发展壮大。继续加大资金投入，打通城市与园区连接瓶颈，完善园区城市服务功能，加快形成产业集聚优势，着力打造一批百亿元工业园区。培育县域工业新增长点，启动北部湾华侨投资区、南间经济带、六钦工业带和一批重点工业镇工业集聚区规划建设。继续实施“抓大壮小扶微”工程，围绕食品、服装、建材、制药、木业、编织等县域特色产业，加快培育一批骨干企业。建立健全县区领导帮办企业制度，多渠道解决县域工业发展中的土地、资金和管理难题。加大亿元企业和规模以上企业培育力度，力争县域规模以上工业产值达650亿元，新增亿元企业30家、规模以上企业30家。

全力服务高新技术产业发展。全面实施“高新产业培育工程”，依托高新区和其他各类开发区，积极布局电子信息、新能源、新材料、节能环保、生物医药、精密制造等产业。做大做强坭兴陶特色产业，着力在陶瓷新材料、创意设计和窑变技术环节进行攻关。创新园区管理体制，积极组建开发投资公司，推进高新区二期开发。继续推进“发明专利倍增计划”，加强高新技术企业申报工作。实施“金种子”企业培育工程，加快推进高新技术企业做大做强。加强领军型人才“520”计划和“筑巢引博”行动，建立健全产学研合作的体制机制，推进科技孵化基地和技术平台建设，增强高新技术产业发展的支撑力。

全力服务非公经济加快发展。非公有制经济和公有制经济一样，都是社会主义市场经济的重要组成部分。必须进一步解放思想，彻底摈弃制约民营经济发展的各种阻力和障碍。坚持“思想上放心放胆、工作上放手放开、政策上放宽放活”的方针，加快出台鼓励和支持民营经济发展的政策意见，充分释放民营经济的发展活力。培育重点建筑企业，推动建筑业做大做强。

二、深入开展“项目推进和招商引资攻坚年”活动，进一步增强经济发展后劲和活力。必须把项目投资作为调结构、增后劲、促发展的重要支撑，继续保持投资适度较快增长，始终不渝地抓好招商引资工作，以大招商带动大建设大发展。

坚定不移推进重大项目建设。坚持和完善项目投资责任制，强化跟踪督查和考核激励机制，加快推动一批重大项目开工、投产。力争国投电厂二期、中电投热电联供一期等项目获国家核准并全面开工建设，争取汽车项目落地，推进中船钦州基地一期、玉柴石化二期、30万吨级支航道、贵港至合浦高速公路等项目全面施工，力促澄星磷化工二期等项目开工。开展中石油二期预可研，推进华谊煤基多联产、华谊氯碱等项目前期工作。突出抓好县区重大项目建设，全年实施亿元建设项目300个以上。

坚定不移地开展全员招商。坚持招商引资、招商选资和招商引智相结合，建立完善招商项目预评估制度，加快引进一批技术水平高、带动能力强、税收贡献大的项目。认真研究新一轮产业转移的特点和规律，加快建立招商引资“企业库、项目库、政策库”，既重视引进国有企业，也重视引进民营资本，务实推进与粤港澳、东部沿海地区和内陆省份的产业合作。建立市领导联系在谈重大招商项目推进机制，强化部门和专业招商团队责任，围绕石化、装备制造等临港产业和高新产业发展，积极开展产业链招商，力争在关键领域和行业重大项目引进上有新突破，实现重大项目“储备一批、引进一批、开工一批、投产一批”的良性循环。

坚定不移抓好要素保障。实施土地、资金、劳动力等生产要素倾斜政策，下决心解决一批现有项目无法落地问题。全面推进城乡建设用地增减挂钩、低丘缓坡和小微企业综合开发利用，积极实施征转分离、区位调整政策，推进土地集约节约开发，多渠道化解用地指标紧缺。进一步强化征地搬迁工作，坚决遏制各种违法抢建现象。推进“引银入钦”向纵深层次推进，加大中小金融机构、准金融企业和创新金融产品的引进工作力度。谋划成立市本级商业银行和担保公司，积极争取中央预算内投资及自治区补助资金。切实加强银企对接，争取开投集团、滨海置业、临海公司企业债券获核准发行。实施企业家培养计划，加大劳动力培训力度，鼓励企业与大中专院校建立人才培养实训基地。

三、深入开展“城市建设年”活动，努力提高城镇化建设质量和水平。认真贯彻落实中央和自治区城镇化工作会议精神，突出抓好滨海新城开发建设，统筹推进城乡协调发展，实现新城新形象、老城有新样、乡镇变模样，力争城镇化率达到46%。

大力推进城市新区开发建设。整合各种有利资源，创新开发体制机制，推动滨海新城开发建设出新形象、新效果。加大基础设施投入，完善功能性项目布局，建成北部湾国际大酒店、东盟生态园等标志性项目，力争建成北部湾大学滨海校区一期，完成沙井岛东岸岸线整治，加快建设北部湾国际人才创业基地、茅尾海综合整治一期工程，争取落实一批新建龙头项目。坚持产业和城市融合发展，加强开发园区和城区功能整合，积极推动高新综合服务配套区、钦州港行政商务新区和三娘湾旅游度假区建设，打造产城融合示范区。启动实施“城市营销”计划，强化城市品牌推广力度，逐步打响钦州的生态宜居内涵和独特文化魅力，促进人口集聚和产业发展。

着力提升城市经营和管理水平。继续大力开展城市环境综合整治，突出市容市貌治理，规划建设物流、公交、停车场等站场，实施城市公交提升工程。推进智慧城市和数字城管建设。统筹推进“三城”创建，深入实施“园林生活十年计划”，完成中山公园改扩建、江滨公园建设，完善白石湖公园、林湖公园等特色城市景观设施，加快城区第二水源地建设，开工建设钦江五桥、环城北路，建成环城东路，打通主城区城市环路，建成黎合江交叉口综合整治项目。加大旧城区改造力度，推进人民路、中山路等历史街区保护规划、更新改造。切实解决社区公共服务用房问题。结合人防工程建设，加快城市地下空间规划及综合利用。

推进县城和重点镇规划建设。积极推进城乡一体化发展，推动公共服务、基础设施、社会管理、规划向县城、乡镇及城乡结合部延伸。编制实施新型城镇化发展规划，继续推进镇村规划集中行动，力争完成所有镇总规和重点镇控规编制。推进重点城镇路网、给排水、污水处理设施建设，抓好管道燃气县县通接入工程。加快发展一批经济强镇、特色小镇、安居村落，努力保护好乡村自然风貌。推动有条件的县开展“撤县设市”申报工作，继续推进灵山江北新区、浦北金浦新区、钦北新城开发建设。加强“镇改办、村改居”工作，稳步推进农民市民化进程。

四、扎实抓好“特色农业提升工程”，加快构建现代高效农业生产体系。全面深化农村经济体制改革，加快构建新型农业经营体系，推进特色农业规模化、标准化经营，多渠道增加农民收入，促进农村经济社会发展。

提高农业现代化水平。切实强化粮食安全意识，推进粮食稳定生产和特色优势农业发展。实施“米袋子”、“菜篮子”升级工程，推进特色优势农业标准化生产，扩大蔬菜、木薯、茶叶等经济作物种植。加快台湾农民创业园、 “中国龟谷”和浦北苗木产业园建设，开工建设龙门港水产产业园等农产品深加工流通项目。大力推进火龙果等优质农产品基地建设，加强农产品批发和物流园区建设，促进农产品顺产顺销。不断加大农业科技投入，建设农业科技重点示范县。积极发展农业合作经济，重点扶持一批农业产业化龙头企业发展。

创新农业经营体制机制。认真落实强农惠农富农政策，加快建立以农户家庭经营为基础、合作与联合为纽带、社会化服务为支撑的新型农业经营体系。大力发展家庭经营、集体经营、合作经营和企业经营，推进特色优势农产品深加工，提高农业产业化经营效益。鼓励土地承包经营权流转，支持农民以经营权入股发展农业产业化经营。实施“农民人均纯收入倍增计划”，积极发展林下经济，推进灵山创建经济强县、灵山和浦北国家西部地区农民创业试点县建设，多渠道增加农民经营性、财产性收入。加大产业扶贫力度，完善扶贫长效机制。

改善农村生产生活条件。继续推进“县镇村道路通畅三年大会战”，实现100%行政村及50%自然村通水泥路。加强农田水利建设，完成病险水库除险加固、中小河流治理、标准海堤建设、渠道防渗等各项任务。完善基层农技推广、信息化服务体系。实施好惠及20万人的农村饮水安全工程。

五、加快建设区域性国际航运物流中心，充分发挥港口辐射带动作用。围绕把钦州港建设成面向中国一东盟合作的区域性国际航运物流中心，抓住建设21世纪“海上丝绸之路”的机遇，强化集疏运体系和大通关能力建设，加快集装箱干线港建设，全面建成亿吨大港。

推进深水航道和大能力泊位建设。建成30万吨级油码头栈桥、支航道，30万吨级航道及油码头投入使用。加快国投煤炭码头、金鼓江石化码头建设，力争建成大榄坪北1－3#泊位，加快推进金鼓江航道建设，开工建设鹰岭作业区3－4#泊位。启动大环10万吨级集装箱码头、5万吨级通用泊位及航道前期工作，完成5亿吨大港规划和老人沙港区规划。港口吞吐能力达到1.1亿吨，力争吞吐量突破7000万吨，集装箱突破80万标箱。

加快完善综合集疏运体系。重点建设对接外部铁路大通道的市域铁路网络，优化整合港口铁路专支线及车场资源，建成运营黎钦铁路复线、大榄坪至保税港区铁路，开工建设火车东站经中马钦州产业园区至三墩铁路专线。加快建设配套站场和货场设施，开工建设钦州港站、马皇站等扩能改造工程项目。加快建设钦灵一级公路及火车东站综合客运枢纽，开工建设沿海高速公路改扩建钦州段、南宁至钦州二级公路改扩建项目，加快浦北至中马钦州产业园区一级公路等项目前期工作。

提升港航综合服务水平。规划建设港航服务业集聚区，争取国家给予更多政策支持。优化内外贸航线网络，新增和加密至东盟国家主要港口集装箱班轮航线，开展区域内贸、水水中转业务及内外贸同港同船运输作业，运营好北部湾三港 “穿梭巴士”，推动集装箱货物中转集聚钦州港。加大外贸集装箱货源培育力度。积极建设“无水港”，扩大与西南中南内陆腹地的海铁联运业务。出台“大通关”扶持政策，完善口岸基础设施，加快建设电子口岸、互联互通电子信息平台，设立航运服务中心，建立口岸和港航工作定期会商联动机制。加快钦州港与东盟国家主要港口城市互联互通，力争完成中国－东盟港口城市合作网络钦州基地首批项目建设。

六、全面深化改革、扩大开放，积极打造对内、对外开放合作新高地。坚持把改革创新、扩大开放贯穿到经济社会发展的各个领域、各个环节，从钦州实际出发，从存在问题入手，坚决破除影响跨越发展的体制机制，进一步增强发展的内生动力。

推进重点领域改革。深化经济体制改革，突出抓好新一轮市直国有企业改制、财税体制、投融资体制、园区体制、科技体制、华侨农林场体制等改革。积极发展混合所有制经济，按照“非禁即准”的原则，鼓励引导民间投资进入市政公用事业、保障性住房、医疗、教育等公共服务领域，研究制订公共资源特许经营管理办法。深入推进“财源经济发展三年倍增计划”，完善上下联动、部门协作抓财源建设的工作机制，继续推动出台行业培植财源扶持政策。深入研究用好用足沿边金融综合改革试验区政策，争取国家、自治区支持钦州在金融、行政管理、对外开放等领域先行先试。完善行政审批配套制度，研究投资准入、审批权下放等重大政策。全面推进自治区级统筹城乡综合配套改革试点，以滨海新城、部分重点镇等区域先行试点，稳步推进户籍制度等改革，探索城乡一体化发展的途径和模式。

扩大对内对外开放。围绕打造西南中南开放发展新的战略支点的“脊梁”，争取国家重大项目和重大政策支持。进一步放大各类开放开发平台的政策效应，积极研究申报设立钦州自由贸易港区。建立健全中马两国政府合作机制，完善“两国双园”联合理事会制度，加快建成中马双边合作的“旗舰”项目。争取尽快出台中马钦州产业园区优惠政策和园区管理办法，洽谈引进中马“硅谷”等项目。拓展钦州保税港区服务功能，加快创建全国进口酒类综合服务产业知名品牌示范区，申报建设进口肉类、水果指定口岸。加快川桂临海产业园建设，带动对内开放合作跨上新台阶。

促进对外贸易稳定增长。发挥港口对产业和贸易的带动作用，力争编织工艺、茧丝绸申报成为自治区级出口转型升级示范基地。申报国家级磷酸产业出口基地，培育电子、农机、粮油等支柱产业出口基地。积极扩大边贸业务。不断加大国际市场开拓力度，组织企业参加中国—东盟博览会等各种境内外知名展会，扩大进出口规模。争取国家尽快批准给予广西中石油国际事业公司原油非国营贸易进口资质。

七、充分发挥沿海港口和独特区位优势，加快构建北部湾现代服务业中心。着力完善港口和城市服务功能，以高速铁路开通为契机，加快布局一批文化、旅游、商贸、物流等服务业项目，推进现代服务业集聚化发展。

推动旅游文化融合发展。围绕规划建设北部湾旅游文化产业发展高地的目标，研究出台扶持旅游文化产业发展的政策措施，着力打造一批旅游文化品牌。加快推进麻蓝岛文化旅游度假区、千年古陶城和北部湾博物馆建设，启动三娘湾旅游区创建国家5a景区。积极开展旅游产品营销，围绕滨海旅游、山水旅游、文化旅游和老城旅游，着力打造一批精品旅游线路。挖掘开发历史文化遗产，开展跳岭头申报国家级非物质文化遗产保护名录，启动坭兴陶烧制技艺申报世界非物质文化遗产。

繁荣发展商贸物流业。加快制定出台促进商贸流通业发展的扶持政策，引进红星·美凯龙等区域性综合商贸企业，培育打造商贸新品牌。推进专业市场建设和农贸市场改造整治，建成东盟农产品大市场一期、东盟商贸城一期小商品城，加快建设北美商贸城一期，开工建设北部湾国际汽车城等项目。启动一批重点镇商贸中心建设。积极发展总部经济、文化创意、电子商务等新兴业态，大力发展休闲养老、健康养生等生活性服务业。加快培育发展现代物流、金融保险、信息服务等生产性服务业。

规划建设一批服务业集聚区。要像发展工业园区一样，支持和引导服务业集聚区加快发展。重点围绕满足城乡居民需求和产业发展需要，调整优化城市和县城商业街区布局，完善工业园区配套服务功能，规划建设新的生产和生活性服务业集聚区。积极推进白石湖中央商务区、沙井岛体育文化体验区、钦州港航运服务集聚区建设。改造提升金海湾大街、人民路、钦州湾广场等综合性商贸服务业集聚区，支持年年丰、恒基广场等城市综合体开发建设，规划建设河东高新城商业圈、火车东站商业集聚区、汽车北站商贸服务区等。依托三级甲等医院，积极规划发展医疗服务和健康产业集聚区。

八、切实保障和改善人民生活，更大力度推进美丽钦州、和谐社会建设。把保障和改善民生放在更加突出位置，为民多办实事好事，积极创新社会管理，构建符合时代特征、满足群众需求的社会保障和管理体系，让发展成果更多、更公平惠及百姓。

加快完善公共服务网。坚持教育优先发展，切实加大教育投入，促进各级各类教育协调发展。加快北部湾大学建设，启动广西英华国际职业学院“专升本”，推进广西医科大学钦州项目、广西安全技术职业学院迁建。开展中小学标准化建设，新建或改扩建一批中小学校及幼儿园，逐步解决大班额问题，加快农村中小学教师周转房建设。实施新一轮职业教育攻坚五年计划，建成市职教中心二期。推进老年大学建设。深化医药卫生体制改革，巩固完善基本药物制度和基层运行新机制，建成市艾滋病防治专区。开工建设市儿童医院、浦北中医院搬迁项目，提高基本公共卫生服务水平。全面完成村级阵地“八个一”建设任务。实施文化惠民工程、市民文明素质提升工程，广泛开展群众性文化体育活动，组织承办广西第十三届少数民族传统体育运动会。

努力健全社会保障网。继续抓好一批为民办实事工程，切实解决好群众关心的热点和难点问题。突出抓好灵活就业人员、个体工商户和非公经济企业社会保险扩面征缴。加大就业创业帮扶，全年城镇新增就业人数2.5万人，转移农村劳动力6万人。加快保障性住房建设，建成各类保障性住房4147套。完善失地失海农渔民社会养老保险政策。加强城乡低保、医疗救助、农村五保供养等工作，实现“应保尽保”，提高社会救助水平。

切实强化社会安全网。加强综治基层基础建设，实现村级综治“五个一”、社区网格化管理、综治信息化系统县镇村全覆盖。加大对社会治安重点地区和“两抢一盗”、黄赌毒等突出问题整治。健全重大决策社会稳定风险评估和突发事件预警、应急处置机制，增强应对债务风险和预防、应对各类突发事件的能力。加强互联网监管，及时回应群众关切，正确引导舆论。创新信访工作机制，构建综治信访便民服务体系，积极排查化解各类矛盾纠纷。推进食品药品监管体制改革试点，完善市、县（区）、镇（街道）食品药品监督管理机构。坚守安全生产“红线”，突出抓好危化企业、矿山、烟花爆竹等重点领域安全生产监管。加强防灾减灾和防汛抗旱防台风工作。

同时，我们要以开展“美丽钦州·清洁乡村”活动为契机，推进农村垃圾、污水处理等基础设施建设，全面推进生态文明建设，保护好青山绿水、蓝天碧海。强化环境保护责任制，重点抓好茅尾海、钦江流域污染防治，坚决完成节能减排目标任务，加强造林绿化。统筹做好人口与计生工作，重视双拥和优抚安置，推进实施质量兴市战略，加强国防动员、民兵预备役、人民防空建设，加大打击走私力度。做好村两委换届工作，支持工会、共青团、妇联等人民团体工作，推动民族、宗教、外事、侨务、科普、新闻出版、统计、海防、地震、农机、气象、水文、档案、地方志、行政学院、老龄、残疾人和关心下一代等各项事业全面进步。

九、下决心转变政府机关工作作风，努力建设人民满意政府。坚持执政为民、服务为本，扎实开展群众路线教育实践活动，切实转变政府职能，着力打造亲民高效廉洁政府。

切实强化服务意识，努力打造亲民政府。加强政府治理能力建设，优化政府机构设置、职能配置、工作流程，完善决策权、执行权、监督权既相互制约又相互协调的行政运行机制。始终把老百姓的事情放在心上，努力做到解决群众关心的问题不推诿、不扯皮、不拖延、不走样，让社会满意、让群众放心。积极推进简政放权，强化政府公共服务职能，加快推进公共资源交易平台建设，积极探索公共事务社会化管理。完善公共服务体系，推进“一服务两公开”向基层延伸，做到公共服务不“缺位”，满足人民群众最基本的社会服务需要。

切实强化创新意识，努力打造高效政府。大力推进政府管理创新和效能提升，重点攻坚社会反映强烈的体制机制障碍，敢于先行先试，以更新的理念、更大的力度、更实的举措，推进各项工作落实。各级政府部门要牢固树立大局意识，敢说实话，能办实事，勇于担当，努力形成分工明确、协同推进、一级抓一级、层层抓落实的工作合力。切实强化工作责任制，严格绩效和问责管理，确保政令畅通、权责一致。

切实强化公仆意识，努力打造廉洁政府。认真执行中央“八项规定”，坚决反对和纠正“四风”。加强调查研究，倡导务实求实，力戒空谈浮夸，开短会、讲短话，进一步精简会议、文件。加强行政效能监察，强化督查考核。严格落实党风廉政建设责任制，强化廉政教育与监督，完善惩治和预防腐败体系。深化领导干部经济责任审计，严肃查处违纪违法案件，坚决惩治腐败。密切与人民群众的联系，增进与人民群众的感情。

各位代表，政府的权力来自人民，对人民负责，受人民监督。我们要自觉接受人大及其常委会的依法监督、人民政协的民主监督，认真听取各民主党派、工商联和无党派人士意见，主动接受社会舆论监督和公众监督，健全民主决策咨询机制，坚定不移地推进依法行政、科学行政、高效行政，勇于承担起钦州发展的历史重任。今天的钦州，已经站到了新的起跑线上，我们承载光荣使命，必将铸就辉煌业绩。让我们更加紧密地团结在以习近平同志为总书记的党中央周围，在自治区党委、政府和市委的坚强领导下，紧紧团结和依靠全市人民，解放思想，振奋精神，锐意进取，敢做善成，为加快实现“五年跨越、十年巨变”、打造中南西南开放发展新的战略支点、提前全面建成小康社会而努力奋斗！
